# Chiral Supramolecular Hydrogel Loaded with Dimethyloxalyglycine to Accelerate Chronic Diabetic Wound Healing by Promoting Cell Proliferation and Angiogenesis

**DOI:** 10.3390/gels8070437

**Published:** 2022-07-13

**Authors:** Yubo Zhang, Weijie Cai, Zun Ren, Yuxiang Lu, Musha Hamushan, Pengfei Cheng, Zhengyu Xu, Hao Shen, Changli Zhao, Pei Han, Wanrun Zhong

**Affiliations:** 1 Orthopedic Department, Shanghai Jiao Tong University Affiliated Sixth People’s Hospital, Shanghai 200233, China; zhangyubo806@163.com (Y.Z.); vegecai@sjtu.edu.cn (W.C.); rzbuer@163.com (Z.R.); 16301020013@fudan.edu.cn (Y.L.); musa127@126.com (M.H.); chengpf@alumni.sjtu.edu.cn (P.C.); xzy_6thhospital@163.com (Z.X.); shenhao7212@shsmu.edu.cn (H.S.); 2 School of Materials Science and Engineering, Shanghai Jiao Tong University, Shanghai 200240, China

**Keywords:** supramolecular hydrogel, chirality, endothelial cells, wound healing, DMOG

## Abstract

Chronic refractory wounds are one of the most serious complications of diabetes, and the effects of common treatments are limited. Chiral hydrogel combined with dimethyloxalyglycine (DMOG) as a dressing is a promising strategy for the treatment of chronic wounds. In this research, we have developed a DMOG-loaded supramolecular chiral amino-acid-derivative hydrogel for wound dressings for full-thickness skin regeneration of chronic wounds. The properties of the materials, the ability of sustained release drugs, and the ability to promote angiogenesis were tested in vitro, and the regeneration rate and repair ability of full-thickness skin were tested in vivo. The chiral hydrogel had the ability to release drugs slowly. It can effectively promote cell migration and angiogenesis in vitro, and promote full-thickness skin regeneration and angiogenesis in vivo. This work offers a new approach for repairing chronic wounds completely through a supramolecular chiral hydrogel loaded with DMOG.

## 1. Introduction

Chronic refractory wounds are one of the most serious complications of diabetes mellitus. Unlike acute wounds, chronic wound healing usually takes more than eight weeks or does not heal at all [1]. Many patients with diabetic wounds often suffer from long-term hospitalization, elaborate nursing and drugs, multiple surgeries, and even lower-limb amputation, resulting in costly medical expenses and a poor quality of life [2].

Wound healing is a complex process involving a variety of cells, the extracellular matrix and various growth factors throughout the first stage (initial injury and rapid hemostasis), inflammation, proliferation, remodeling, and so on [3]. However, the wound healing of diabetic ulcers does not completely follow the processes above. Under the influence of persistent hyperglycemia and metabolic-related factors, inflammatory cells such as macrophages can produce a lot of inflammatory precursor factors to maintain the state of inflammation [4]. The hyperglycemia environment and the chronic inflammation of non-healing diabetic wounds reduce the number of endothelial cells and weaken their ability to proliferate, migrate, adhere, and synthesize blood vessels by down-regulating the expression of VEGF and angiogenin-1 (ANGl) and its receptors, inhibiting the formation of new capillaries and delaying wound healing [5].

At present, the routine treatment of diabetic refractory wounds mainly includes strengthening clinical nursing, reducing local pressure, improving microcirculation, and controlling inflammatory reactions, but it is difficult to reduce the time of wound healing in clinical application [6]. The use of tissue engineering technology to promote wound healing is one of the most popular treatment strategies. Hydrogels have been widely used to provide three-dimensional (3D) environments that are biophysically similar to the human extracellular matrix (ECM), which cannot be modeled by a traditional 2D matrix [7]. Supramolecular hydrogels have been widely studied as intelligent 3D scaffolds due to their excellent abilities to stimulate the natural ECM and their responsiveness to various external stimuli, such as temperature, redox reagents, ultrasound, and light irradiation [8]. Chirality is one of the most significant biochemical signatures of life and has a great influence on many biological events [9]. Many researchers have shown that the chirality of nanofibrous structures has a great influence on cell proliferation and adhesion in the 3D ECM [10]. The cells were found to adhere and grow significantly more intensively in l-type than in d-type 3D environments [11].

In addition to the beneficial chiral supramolecular hydrogel scaffolds, the chemically active agents are also considered to create a synergistic microenvironment to accelerate diabetic wound healing [12]. Dimethyloxalyglycine (DMOG) is a HIF-PH inhibitor with cell permeability. It can inhibit the effect of HIF-PH and up-regulate the expression of HIF-1α under normal oxygen concentration [13]. HIF-1α is the upstream gene of VEGF. The overexpression of HIF-1α can enhance the secretion of VEGF in endothelial cells. At present, VEGF is recognized as a factor with a strong ability to promote angiogenesis, which can promote the proliferation and differentiation of endothelial cells and induce the formation of neovascularization [14]. In addition, HIF-1α can also enhance the secretion of cytokines involved in the formation of new blood vessels, such as bFGF, ANGPT2, PLGF, and SCF, thus promoting capillary regeneration. Hence, the loading and release of DMOG drugs by chiral supramolecular hydrogels may be a promising strategy to stimulate vascularization in diabetic wound sites [13].

Herein, we design a novel supramolecular chiral hydrogel loaded with DMOG. It is hypothesized that the chirality of the nanofibrous structures combined with the controlled release of DMOG drugs create a synergistic microenvironment that rapidly promotes angiogenesis and efficient healing of diabetic wounds. In this work, hydrogels containing different amounts of DMOG were prepared, and the microstructures and drug-release profiles were evaluated. Moreover, The biological effects of chiral nanofiber structure combined with DMOG on the growth of human umbilical vein endothelial cells (HUVECs) in vitro and diabetic wound healing in vivo were systematically studied, and the mechanism was proposed.

## 2. Results

### 2.1. Fabrication and Analysis of Chiral Supramolecular Hydrogel

**Figure 1:**
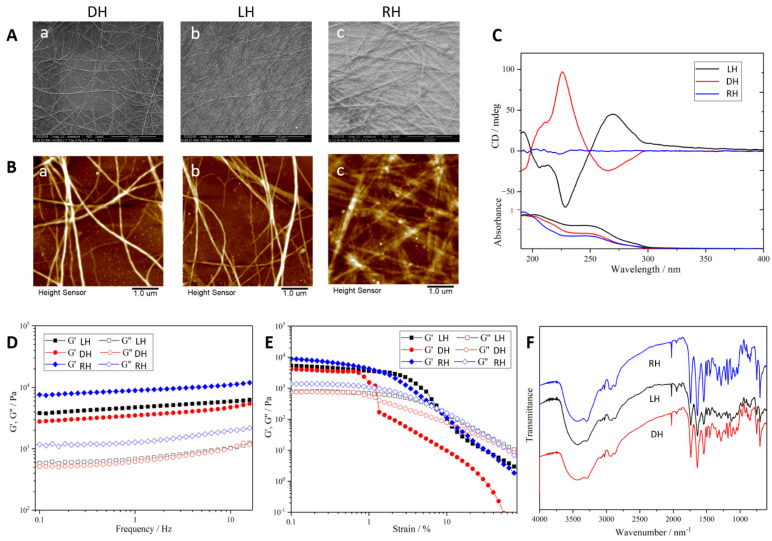
Characterization of chiral supramolecular hydrogels. (**A**) SEM images of different chiral supramolecular hydrogels. DH (a), LH (b), RH (c), scale bar = 10 μm; (**B**) AFM image of different chiral supramolecular hydrogels. DH (a), LH (b), RH (c), scale bar = 1 μm; (**C**) CD spectroscopy; (**D**) FTIR spectra; (**E**,**F**) rheology of hydrogels assessed by rheometer.

The chiral supramolecular hydrogel was fabricated by two enantiomers (l-ph and d-ph) in a self-assembly strategy as shown in Figure 1. The chirality of the hydrogel was characterized by SEM, AFM, and CD kinetic spectra. The SEM images displayed the morphology of the hydrogel, which demonstrated that the opposite chiral nanofibers constructed LH and DH hydrogels, while the RH hydrogel contained nanofibers without chirality. Opposite helical nanofibers in the LH and DH hydrogels were also shown by AFM. A mirror-image relationship was also observed between the LH and DH hydrogels in the CD kinetic spectra (Figure 1A–C). However, the RH hydrogel did not display CD signals. These data indicated that it was the self-assembled helical nanofibers, not the individual monomers, controlling the chirality of this supramolecular hydrogel—which has been shown in previous research. Despite the differences between their chiral nanostructures, the LH, DH, and RH hydrogels showed quite similar chemical and physical properties as measured via FTIR spectrometer and rheometer (Figure 1D–F). These measurements allowed us to assess the effects of supramolecular chirality on the cellular behavior of endothelial cells and wound healing.

### 2.2. The Release of DMOG from Hydrogel

**Figure 2:**
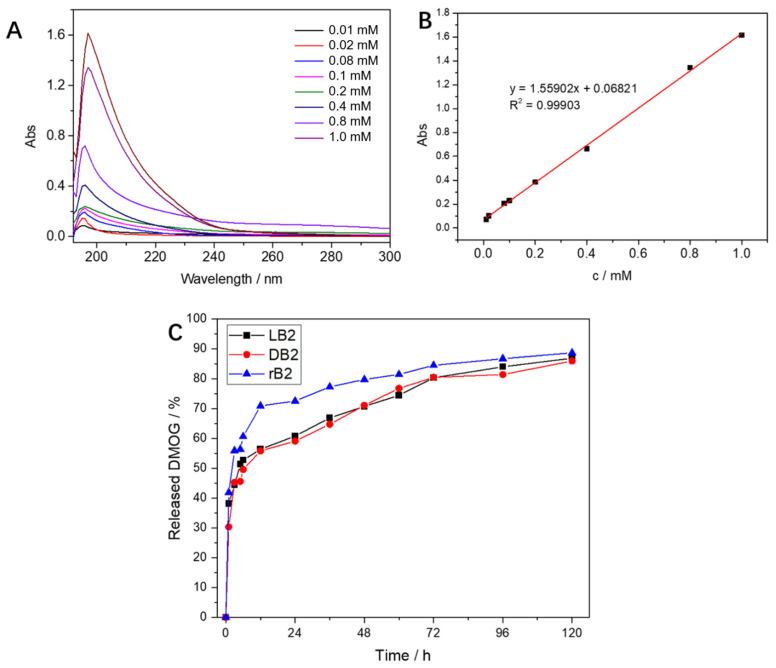
(**A**) The absorption curves of different concentrations of DMOG at the full wavelength, all have absorption peaks at 198 nm; (**B**) the regression-curve fitting of the DMOG concentration and the absorbance at a wavelength of 198 nm was performed, and it was found that the correlation coefficient R^2^ = 0.99903, the correlation was good; (**C**) after mixing DMOG with the chiral hydrogels, the absorbance test was carried out at a 198 nm wavelength. The mixed-group hydrogel could release 70% in the first 12 h, while the left-handed and right-handed chiral hydrogels released slowly; after 120 h, almost complete release was observed.

To examine the rate and ratio of drug release in the hydrogels, we measured the drug concentration using UV spectrophotometry. First, we determined the peak of the absorption wavelength of the drug at 198 nm with a series of standard concentrations of the DMOG drug (Figure 2A). A good correlation between concentration and absorbance was found using standard curve regression, with R^2^ = 0.99903 (Figure 2B). Therefore, we performed absorbance measurements on DMOG-loaded hydrogels at a wavelength of 198 nm. We found that within the first 24 h, drug release was faster in the mixed group, about 70%, while it was only about 59% in the LH and DH hydrogels. Drug release tended to be slow over the next time period, until no further increase in release occurred after 120 h (Figure 2C).

### 2.3. Effects of DMOG, Hydrogel, and Combination of DMOG and Hydrogel on Cell Proliferation, Migration, and Tube Formation

**Figure 3:**
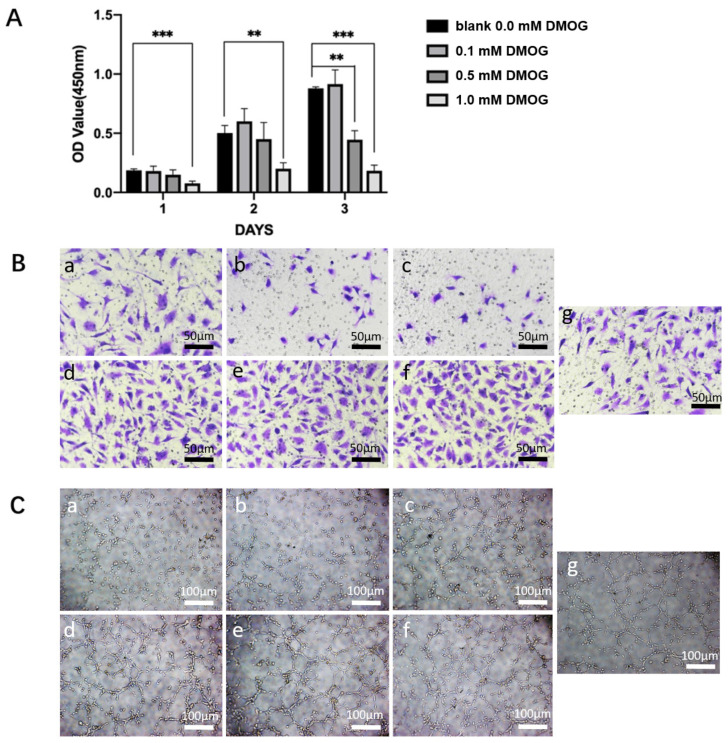
(**A**) Effects of different concentrations of DMOG on HUVECs. Compared with the control group, 0.1 mM DMOG can promote cell proliferation; while 0.5 mM and 1 mM DMOG can significantly inhibit cell viability. (**) = *p* < 0.01, (***) = *p* < 0.001. (**B**) effects of different chiral hydrogels (**a**: LH, **b**: DH, **c**: RH) and three different chiral hydrogels containing DMOG (**d**: LH-DMOG, **e**: DH-DMOG, **f**: RH-DMOG) on the migration ability of HUVECs. Compared with the control group (**g**), the hydrogels containing DMOG can significantly promote the cell migration rate; (**C**) effects of different chiral hydrogels (**a**: LH, **b**: DH, **c**: RH) and three different chiral hydrogels containing DMOG (**d**: LH-DMOG, **e**: DH-DMOG, **f**: RH-DMOG) on the tube-forming ability of HUVECs. Compared with the control group (**g**), the hydrogels containing DMOG can significantly promote the ability of endothelial cells to form tubes, and the number of lumens and branches increases significantly.

After different concentrations of DMOG drug pairs were added to endothelial cells, CCK-8 experiments were performed on days 1, 3, and 5, respectively. The ECM without DMOG was used as a blank group. We found that 0.1 mM DMOG can promote cell growth, while 0.5 mM and 1 mM DMOG have obvious inhibitory and toxic effects on cell growth (Figure 3A). Therefore, we used 0.5 mM DMOG to mix with the hydrogels in subsequent experiments.

In cell migration experiments, we found that—compared with the control group—cell migration was similar in the LH group, while cell migration was inhibited in the DH and RH groups. While different chiral hydrogels (whether DH, LH, or RH) containing DMOG enabled more endothelial cells to migrate to the lower chamber, (Figure 3B). In tube-formation experiments, We found the RH group and the control group had a similar tubule-forming ability, while the LH and DH tubule-forming ability were slightly weaker. Different chiral hydrogels (whether DH, LH, or RH) containing DMOG enhanced the ability of endothelial cells to form tubular structures (Figure 3C).

### 2.4. Skin Wound General Observation and Wound Closure Calculation

**Figure 4:**
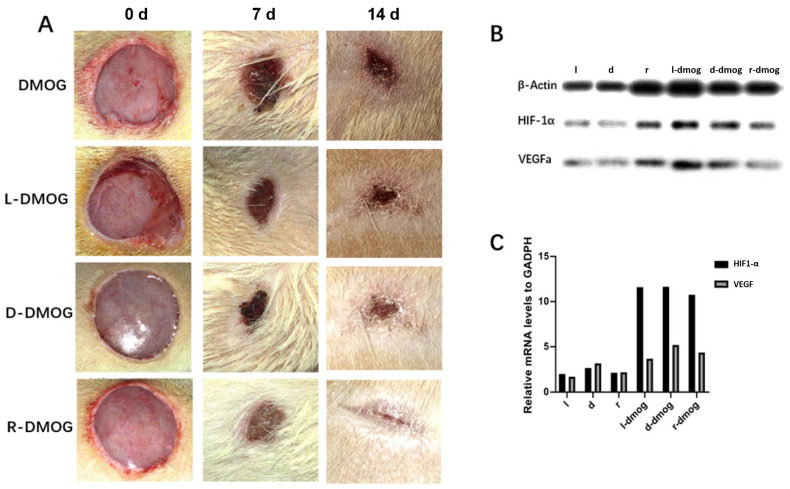
(**A**) Skin wound healing in diabetic rats. Compared with the pure DMOG drug, the use of the hydrogel compound drug can significantly promote the wound-healing rate of diabetic rats; (**B**) the protein was extracted from the wound of the rat, and it was found that the right-handed chiral hydrogel combined with the DMOG drug group could significantly promote the expression of HIF-1α and VEGF in the tissue; (**C**) the extraction of mRNA from rat skin and qPCR showed that the hydrogel containing DMOG could significantly promote the content of HIF-1α and VEGF in the tissue.

During postoperative wound observation, we found that different chiral hydrogels had an effect on the rate of wound healing. Among them, the DMOG-loaded hydrogel can significantly improve the rate of wound healing (Figure 4A). After protein and mRNA extraction from wounds, we found that right-handed chiral hydrogels combined with DMOG could promote the expression of HIF-1α and VEGF in tissues (Figure 4B,C).

### 2.5. Histology Analysis

**Figure 5:**
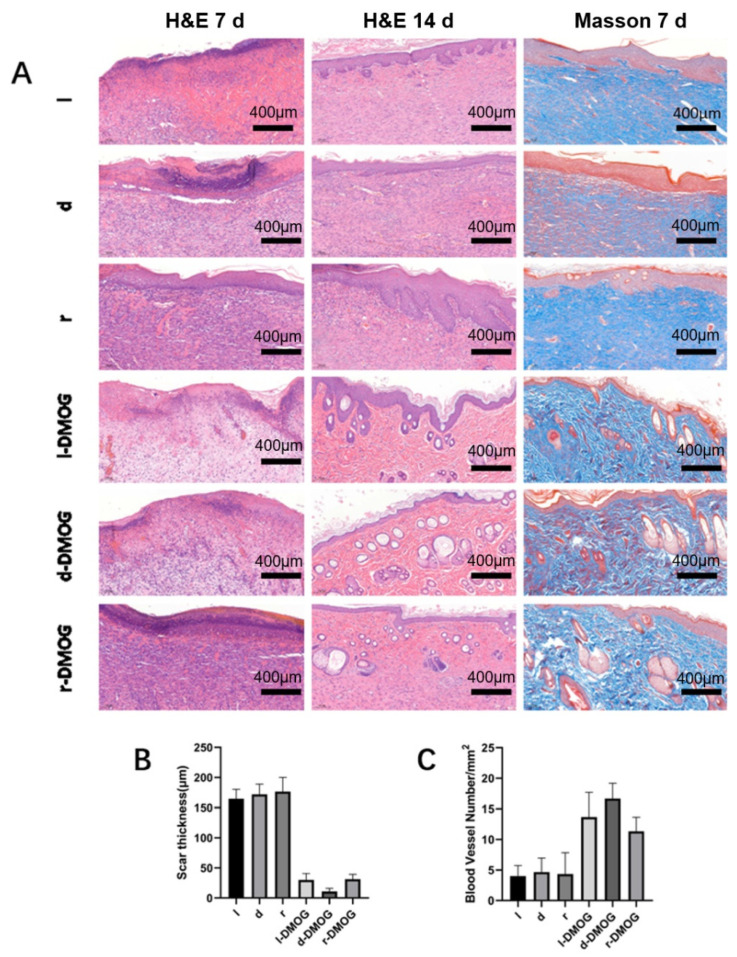
(**A**) H&E staining and Masson staining were performed on the wounds of different groups of rats; (**B**) the scar thickness was measured, and it was found that the hydrogel containing the drug group could significantly reduce the scar thickness; (**C**) counting the number of subcutaneous new blood vessels demonstrated that the hydrogel containing DMOG can significantly increase the content of new subcutaneous blood vessels. (Scale bar 400 μm).

At 7 and 14 days after operation, the wounds of rats were observed by H&E staining (Figure 5A). We found that different hydrogels containing the drug were able to significantly reduce the thickness of the local scar (Figure 5B). At the same time, Masson staining was performed on the subcutaneous tissue, and it was found that in the hydrogel group containing the drug, the subcutaneous collagen tissue was arranged in a more orderly manner, and the number of new blood vessels increased significantly (Figure 5C).

## 3. Discussion

Diabetes is a universal worldwide public health problem [15]. The number of people with diabetes in the world was 415 million in 2015 and will reach 642 million by 2040. Diabetic wound healing is poor due to neutrophil activation, fibroblast migration disorder, and morbid angiogenesis [16]. At present, the main treatments for diabetic wounds include dermal substitutes developed in the laboratory, stem cell therapy, and recombinant growth factors at higher than physiological concentrations [17]. However, these treatments are difficult to maintain and can have side effects [18]. Successful strategies for the treatment of chronic refractory trauma include the promotion of wound angiogenesis, collagen deposition, and the completion of epidermal revascularization. In this experiment, the skin wounds of diabetic rats were treated with DMOG combined with a chiral hydrogel, and the healing speed was faster than that of a simple chiral hydrogel. H&E staining showed that a DMOG-hydrogel could effectively induce the proliferation of vascular endothelial cells at the wound site and complete revascularization. The collagen arrangement was more orderly in groups containing DMOG.

DMOG has the ability to up-regulate the expression of HIF-1α, which effectively promotes angiogenesis and makes it an ideal choice to promote wound healing [19]. In addition, it has the advantage of a good biosafety and record of clinical application [20]. However, the application of the DMOG molecule is limited because of its unstable properties and low bioavailability in vivo. In this experiment, a chiral hydrogel combined with DMOG was used to promote the healing of chronic refractory injuries.

HIF-1 is the core regulator of intracellular hypoxia-induced responses in hypoxic environments [21]. HIF-l plays an important role in hypoxia caused by ischemia, which is mainly regulated by the HIF-lα subunit. During hypoxia, cytokine release, hormone regulation, and genetic changes, HIF-lα is transferred from the cytoplasm to the nucleus, where it dimerizes with HIF-lβ subunit and then binds to the promoters of corresponding genes such as VEGF and VEGFR2 to promote their expression [22]. This factor plays an important role in improving the function of EPCs and promoting neovascularization [23]. At the same time, HIF-1α is the upstream gene of VEGF. The overexpression of HIF-1α can enhance the secretion of VEGF in endothelial cells. VEGF is currently recognized as a strong angiogenic factor, which can promote the proliferation and differentiation of endothelial cells and induce neovascularization [24]. In addition, HIF-1α can also enhance the secretion of cytokines involved in the formation of new blood vessels, such as bFGF, ANGPT2, PLGF, SCF, etc., thus promoting capillary regeneration.

Supramolecular hydrogels self-assemble low-molecular-weight gel factors into hydrogels with various micro-and nanostructures through non-covalent interactions such as hydrophobic interactions, hydrogen bonding, and π-π interactions, and their composition and mode of action are closer to the natural extracellular matrix, so they have a better biocompatibility and good drug-loading characteristics, as well as stable, long-acting, and controllable drug-release characteristics [25]. It has been proven that hydrogels can promote wound healing in rats [26]. In this experimental study, the DMOG-hydrogel showed more wound-healing activity than the chiral hydrogel alone. Part of the reason may be the continuous release of DMOG to maximize the use of its pharmacologically-active substances, and another reason may be the role of chiral hydrogel components in wound healing. Hydrogel can accelerate wound healing because it can provide a moist microenvironment for skin wounds, absorb wound exudates, and protect wounds from infection [27]. The use of hydrogel as a drug-delivery system, loaded with DMOG, strengthens the role of hydrogel in promoting wound healing.

## 4. Conclusions

In this study, we prepared different chiral hydrogels containing DMOG, measured the drug-release profiles in them, and applied the composite hydrogels to the wounds of diabetic rats. We found that the drug was released more slowly in the chiral hydrogel, and the supramolecular chiral hydrogel of the composite drug could significantly promote wound healing in rats and reduce the thickness of the scar. This effect may be through the HIF-1α–VEGF pathway. Through cell experiments, we further confirmed that the chiral hydrogel containing DMOG can promote the proliferation, migration, and tube-forming ability of endothelial cells, and further confirmed that its mechanism of promoting wound healing may be related to the promotion of angiogenesis. These results suggest that the chiral hydrogel containing DMOG can be used as a novel wound dressing for the treatment of chronic diabetic wounds and to promote the healing of chronic wounds.

## 5. Materials and Methods

### 5.1. Materials

**Figure 6:**
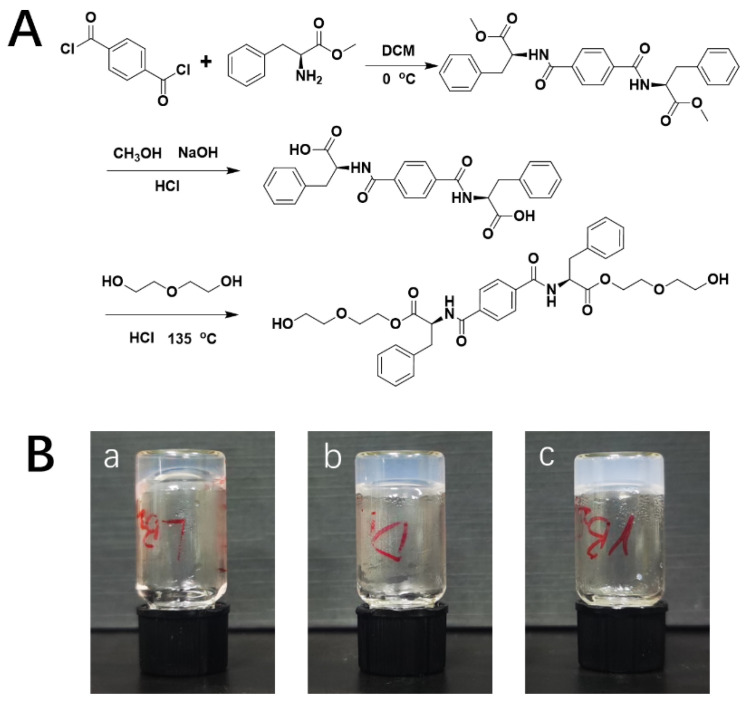
(**A**) Synthesis roadmap of hydrogelators; (**B**) images of LH (**a**), DH (**b**), and RH (**c**) hydrogels.

The hydrogelators were synthesized by the methods presented in previous studies [28,29]. Briefly, a solution of 2.44 g of 1,4-phthaloyl chloride (12 mmol) in 20 mL of dichloromethane was incorporated drop-by-drop to 100 mL of a dichloromethane solution comprising 5.39 g of L/D-phenylalanine methyl ester hydrochloride (25 mmol) plus 4.2 mL triethylamine (30 mmol) in an ice-water bath. The solvents underwent overnight stirring and then were roto-evaporated. The remainder was next dissolved in methanol. Then, 10 mL of NaOH (2 mol/L) was incorporated and stirred for 24 h until the preparation was clear. The solvents were again roto-evaporated, and the remainder was dissolved in DI water. The solution was then acidized with HCl (3.0 M) to adjust pH to <3.0. The precipitates were then filtered and dried. The white powder was then dissolved in diethylene glycol at 135 °C in an oil bath for 4 h, and the mixture was poured into ice water (Figure 6A). Gel-like precipitates were gathered and dried in a vacuum oven. Finally, LH and DH were collected as white solid powders.

### 5.2. Preparation of Supramolecular Chiral Hydrogels

LH gelator (4 g/L) and different amounts of DMOG were placed in a glass tube and dissolved in deionized water through ultrasound. This preparation was then employed to make hydrogel by applying heat until overall dissolution. Posterior refrigeration was at 25 °C. A successful product was obtained when a uniform solid material appeared in the tube and no gravitational flow happened after inverting the vial (Figure 6B). DH and racemic (RH) hydrogels (identical combination of two enantiomers) were made to evenly assess their properties.

### 5.3. Characterizations of Supramolecular Chiral Hydrogels

Then, instrument and test methods were conducted as presented previously [15]. The nanofibrous hydrogels were characterized by circular dichroism (CD) kinetic spectra (ASCO J-815 CD spectrometer, Tokyo, Japan), scanning electron microscopy (SEM, FEI Quanta 250 microscope), and atomic force microscopy (AFM, E4 Sweep microscope; Seiko, Japan). The chemical properties of hydrogels were measured with a Fourier transform infrared (FTIR) spectrometer (EQUINOX55, Bruker, Munich, Germany). A rotational rheometer (Gemini HR nano, Malvern, UK) was used to measure the rheological properties of the hydrogels.

### 5.4. Controlled Release of DMOG

To determine the DMOG UV-absorbance standard curve, hydrogels were prepared by mixing 1 mM DMOG with gelling factor (4 mg/mL, 6.29 mM). The hydrogel was first immersed in a 37 °C PBS solution and placed on a shaker, before extracting 1 mL of the supernatant at diverse times to test the absorbance and immediately supplementing with 1 mL of PBS. Thus, the DMOG release concentration was calculated.

### 5.5. Study on the Effect of Supramolecular Chiral Hydrogel Loaded with DMOG on the Behavior of HUVEC

The Chinese Academy of Sciences provided the HUVECs. The culture of the dispersed cells was performed using the endothelial culture medium (ECM, Sciencell 1001, Shanghai, China) and placed on a wet incubator (5% CO_2_, 37 °C). Only HUVECs from passages 2–7 were collected for the following experiments.

The cytocompatibility of DMOG (Sigma-Aldrich, Shanghai, China) with HUVECs was tested with CCK-8 assays. Briefly, cell seeding occurred in 96-well plates (5 × 10^3^ cells/well). Afterward, the plates were covered with ECM containing varying amounts of DMOG. On days 1, 3, and 5 of the culture, 100 μL of CCK-8 solution (10%) was incorporated into each well. Then, the cells were let rest for 1 h before absorbance measurement at 450 nm using a UV spectrophotometer. This way, an appropriate DMOG concentration range for the cells was achieved and employed in the next two experiments.

The role of DMOG in the migration of HUVECs was evaluated through a transwell test. For this purpose, the higher chamber of a 24-well transwell plate with pore size = 8 μm (Corning) was used to culture 5 × 10^4^ cells with ECM. In the inferior chamber, extracts (600 μL) taken from diverse clusters of hydrogels and hydrogels with DMOG were deposited. Cell collection from the higher chamber was conducted with a cotton swab after 8 h. Later, cells migrating to the inferior chamber were fixed with paraformaldehyde (4%), continued by crystal violet staining (0.5%, 10 min). Cell observation wais facilitated by optical microscopy (Leica, Wetzlar, Germany)

### 5.6. Tube-Formation Assessment

To assess tube formation, a 48-well plate was filled with Matrigel (100 μL, Becton Dickinson, Andover, MA, USA) and then with gel (37 °C, 30 min). Cells with a density of 3 × 10^4^ cells per well were seeded into the Matrigel. Cell treatment consisted of adding media with extracts from diverse sets of hydrogels (including those with DMOG). After 6 h of incubation, tube formation was assessed through an optical microscope. The angiogenesis features to be noted involved nodes and tubes, signs of the early and advanced phases, respectively. These features were manually detected following the manufacturer’s guidelines. When the proliferation, migration, and tube-formation essays were finished, the precise DMOG concentration was measured.

### 5.7. In Vivo Wound Healing Test

The Animal Care and Experimental Committee of Shanghai (Jiao Tong University-affiliated Sixth People’s Hospital) approved all animal experiments. First, Sprague–Dawley rats (N = 48, males) aged 8 weeks were under observation for 1 week and then starved for a night before diabetic stimulation. Baseline levels of blood sugar were registered. The following morning, diabetic induction was conducted using streptozotocin (65 mg/kg b.w., i.p.). Three days later, blood glucose was determined again. Only those animals with more than 300 mg/dL of blood glucose were designated as experimental subjects. They were under vigilance for two additional weeks before skin wounding.

The excisional model of injury-splinting was conducted according to a previous publication (wound diameter = 20 mm) [29].

A digital camera was employed to record the injury cure through days 0, 7, and 14. The wound surface was determined via an image assessment program (NIH Image). Wound closure (%) was deciphered as:

% wound closure = (A_0_ − A_t_)/A_0_ × 100

A_0_ being the wound area at day 0, and A_t_ being the wound are at days 7 and 14, respectively.

Animal sacrifice took place on days 7 and 14. Samples of skin were taken from the wound surface and at 5 mm from that area. Both kinds of samples underwent fixing in formalin (10%), dehydration in gradient alcohol, embedding in paraffin, and sectioning into 5 μm pieces. After that, Masson’s trichrome and hematoxylin-eosin (H&E) coloring were performed.

The effect of hydrogel on the expression of genes VEGF and HIF-1α was evaluated through quantitative reverse transcription-polymerase chain reaction (qRT-PCR) as suggested by the manufacturer. For this purpose, RNA was extracted from the cell samples treated with different hydrogels by Trizol reagent (Takara, Japan), and further synthesized to cDNA using the M-MLV enzyme (Vazyme, China). Then, the samples were used for qRT-PCR after adding ChamQ SYBR-qPCR Master Mix (Vazyme, China). GAPDH was selected as a normalizer. The relative mRNA levels of VEGF (human) and VEGF (rabbit) underwent normalizing and analyses through the 2^−ΔΔCt^ 21 method. Three individual repeated experiments were performed to calculate the average value.

Wound bed tissue was pulverized by a tissue-grinding machine to extract total proteins. Protein concentration was quantified via the bicinchoninic acid assay (BCA) kit (Beyotime). After subjection to SDS electrophoresis, each protein sample was transferred to a nitrocellulose membrane (0.45 μm, Millipore, St. Louis, MO, US) and blocked. Then, the membrane was let rest with anti-VEGF (Affinity, DF7470; 1:1000) and anti-Collagen I (PTG, 14695-1-AP; 1:1000) at 4 °C, overnight. Antigen-antibody complexes were visualized with enhanced chemiluminescence detection reagent (Millipore), and the signal intensities quantified by ImageJ (version 1.8).

### 5.8. Statistical Assessment

All statistical calculations were obtained via the GraphPad Prism8.0 software. Inter-group statistical discrepancies were weighed by one-way tests of variance first, and then by Dunnett posterior testing. All values are expressed in the mean ± standard deviation (SD) format. Statistical significance was granted when *p* value < 0.05. Asterisks signify: (*) = *p* < 0.05, (**) = *p* < 0.01, (***) = *p* < 0.001.

## Data Availability

The data presented in this study are available on request from the corresponding author.
